# The Restrain Food Database: validation of an open-source database of foods that should be eaten more or less as part of a healthy diet

**DOI:** 10.1098/rsos.220923

**Published:** 2022-11-23

**Authors:** Mark Randle, Ines Duarte, Leah Maizey, Loukia Tzavella, Rachel C. Adams, Christopher D. Chambers

**Affiliations:** Cardiff University Brain Research Imaging Centre (CUBRIC), School of Psychology, Cardiff University, Cardiff CF24 4HQ, UK

**Keywords:** open-source image database, food, food stimuli, healthy diet, personalization

## Abstract

Studies of food-related behaviours often involve measuring responses to pictorial stimuli of foods. Creating these can be burdensome, requiring a significant commitment of time, and with sharing of images for future research constrained by legal copyright restrictions. The Restrain Food Database is an open-source database of 626 images of foods that are categorized as those people could eat more or less of as part of a healthy diet. This paper describes the database and details how to navigate it using our purpose-built R Shiny tool and a pre-registered online validation of a sample of images. A total of 2150 participants provided appetitive ratings, perceptions of nutritional content and ratings of image quality for images from the database. We found support for differences between Food Category on appetitive ratings which were also moderated by state hunger ratings. Findings relating to individual differences in appetite ratings as well as differences between BMI weight categories are also reported. Our findings validate the food categorization in the Restrain Food Database and provide descriptive information for individual images within this investigation. This database should ease the burden of selecting and creating appropriate images for future studies.

## 1. Background

Investigations into eating-related behaviours frequently use tasks involving a variety of food images, that are categorized for the purpose of investigating differences in outcomes (e.g. differences in ratings between high and low energy dense foods) [1,2]. The purpose of these categories is to provide some practical value for quantifying a problem such as gaining a better understanding of the associations between habitual intake of high-calorie energy dense foods and weight- or obesity-related health issues [3].

Energy-dense foods are typically highly desired and inform the subjective experiences of liking and food cravings [4]. These are often processed foods high in fat, salt or sugar with the exception of fruit which has been reported as being commonly craved [5]. The extent to which an individual experiences food cravings seems an important factor for weight-related issues. An increase in frequency of cravings for energy-dense foods is associated with a greater body mass index (BMI) [6]. Increases in age [7] and male relative to female participants demonstrate decreases in food cravings [8,9], though others have reported no differences in cravings between sex [10].

Hunger has also been implemented in hedonic processing of food cues. May *et al.* [11] state that hedonic processing of food cues occur as a result of learned associations between internal (e.g. hunger) and external (e.g. environmental) cues which have previously been paired with subsequent consumption. Once a food-related stimulus has been perceived, more cognitive resources are allocated towards information relevant to the desire. It is further elaborated on through increased accessibility and awareness of food-related information in working memory [12].

However, not everybody will respond the same way to the same food cues; a prominent feature of appetite-related processes is the existence of large inter-individual variability [13]. Failure to account for these individual differences could be a key reason why the predictive utility of cognitive tasks for weight and eating-related behaviours has been unreliable [14]. One approach that accounts for individual differences is the use of personalized stimuli in which the participant is provided the opportunity to choose the foods which will be associated with a particular intervention or measurement [15]. However, collections of images are typically non-standardized and often collated by researchers on a study-by-study basis. This is labour intensive and inefficient at a community level, and thus represents a constraint for many investigations [16].

There are currently five open-source food image databases intended for use in psychological research (for a more detailed overview, see Toet *et al*. [17]), including the FoodCast Research Image Database (FRIDa) [18], Food-pics [19,20], The Open Library of Affective Foods (OLAF) [21,22] and the Full4Health Image Collection (F4H) [23].

The most recently published food image database is CROss-CUltural Food Image Database (CROCUFID) [17] which is a standardized food image database consisting of 675 food and 165 non-food images from Asian and Western cuisines. The database was validated on outcomes relating to recognition, arousal, perceived healthiness and desire to eat in 805 individuals from the UK, US and Japan taking part in an online survey. Foods are categorized as being universal (e.g. fruit and vegetables, etc.), unappealing (e.g. uncooked chicken, spoiled leafy salad, etc.), typical Western foods (e.g. hotdog, burger, etc.) or typical Asian foods (e.g. sushi, rice-bowls, etc.).

The Restrain Project is a wider ongoing investigation into the effectiveness of cognitive training for influencing weight change and various other measures of eating-related behaviour (see https://play.google.com/store/apps/details?id=com.connectinternetsolutions.restrain or https://apps.apple.com/gb/app/restrain/id1480827339). One intended output of this project is to develop and validate an extensive food database that provides participants with a virtual supermarket consisting of the foods that were selected on the basis that participants would want to eat more or less of them as part of a healthier diet.

The Restrain Food Database is an open-source food image database consisting of 626 food items. Broadly, food items are distinguished between ‘Eat More’ (523 items) based on the NHS five-a-day recommendation [24], and ‘Eat Less’ (103 items) based on the Food Standard Agency's traffic light system [25]. Within these categories are further subcategories such as fruits, vegetables, generic/chain takeaway, bakery sweet/savoury, etc. Descriptive information for the sample of images in the current investigation regarding nutritional content (actual and perceived content), subjective ratings (i.e. liking, perceived healthiness, cravings, frequency of consumption) and image quality (i.e. recognizability, quality and valence) are provided to aid decisions when creating study material.

Our database has three main advantages over those currently available, making it well suited for designs utilizing personalized stimuli: (i) our database provides a greater range of food categories (e.g. fruits, vegetables, bakery sweet/savoury, takeaway, etc.); (ii) our database provides multiple exemplar images for each food item varying in aspects of image characteristics such as angle and portion size; (iii) our dedicated R Shiny search tool uses the descriptive information to provide a user-friendly interface to navigate the food database.

This paper is presented in two sections. The first describes the food database and OSF project where these images can be accessed, and how to use our dedicated R Shiny app to find appropriate images and their descriptive information generated in this paper.

The second section describes our attempt at validating the food image database on outcomes relating to appetite, perceived nutritional content and usability of images. We provide descriptive information on differences in ratings between food categories. We empirically validate our categorization of foods as those that you wish to eat more/less of by contrasting ratings of taste, craving and healthiness between food categories. We also investigated whether state levels of hunger and demographic factors (e.g. age, sex, BMI) explained individual differences in the ratings provided during the task.

## 2. The Restrain Food Database

### 2.1. The virtual supermarket

The virtual supermarket (VSM) comprises 626 foods and soft drinks divided into two shops: (i) one in which users are asked to select foods they would like to eat less of (i.e. high in fat, saturated fat, salt and/or sugar; *n* = 523); (ii) one in which they would like to eat more of (i.e. fruit and vegetables; *n* = 103). Each of these foods is associated with nutritional information per 100 g or per ml (kcals, fat, saturated fat, sugar, salt), diet information (vegan, vegetarian, pescetarian) and allergy information (milk, eggs, fish, shellfish, peanuts, tree nuts, wheat, soy).

The ‘Eat More’ foods were strictly limited to fruits and vegetables, where 80 g counts toward the five-a-day recommendation [24]. The ‘Eat Less’ foods were selected based on popularity within UK supermarkets and restaurants and were included based on the Food Standard Agency's traffic light system [25]. Foods were included if they contained at least 2 amber values (per 100 g: sugar >5 g; fat > 3 g; saturated fat >1.5 g; salt >0.3 g) and soft drinks that are at least amber for sugar (>5 g per 100 ml).

Each food is associated with seven exemplar images (e.g. different angle, portion size, etc.). Three food items also have images of gluten-free alternatives. The complete database consists of 4286 images that are freely available to use, with or without modification (each image has a Creative Commons licence, which is a licence that enables free distribution of other people's work). There are 4092 unique images as some food images are duplicated within the database (e.g. some of the same images will be used for crisps within the same brand as differences in flavour cannot be identified visually). These images are all in jpeg format, 400 × 400 pixels and present the food either against a white background or where the food fills the frame.

### 2.2. OSF web page

**Figure 1.**
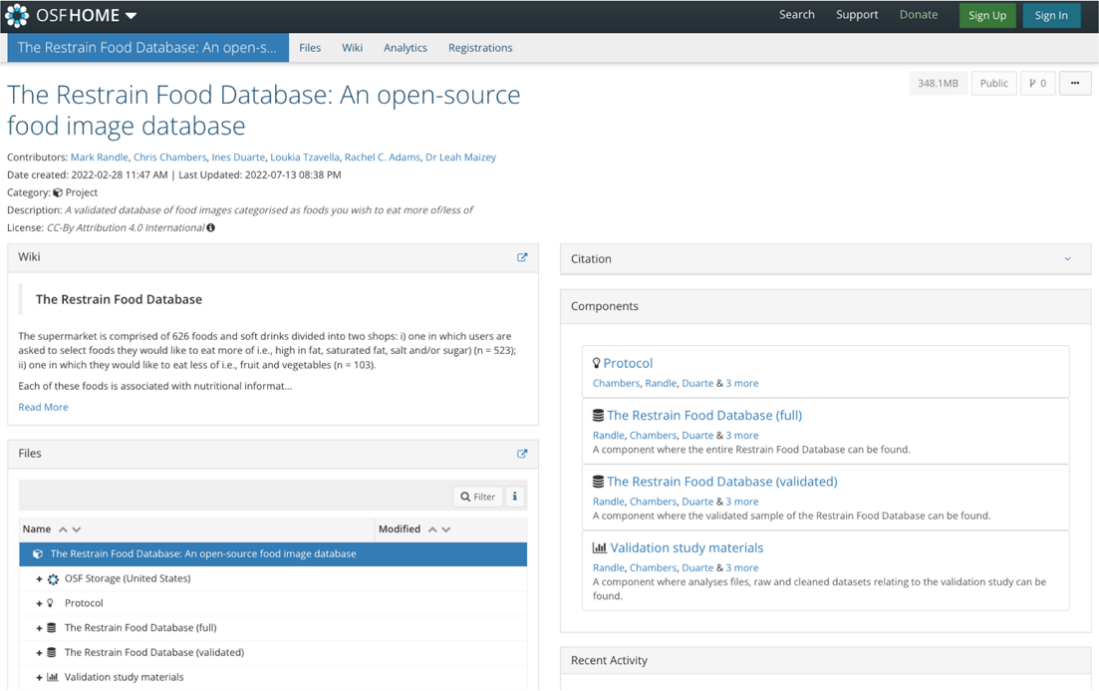
Image showing the homepage of the OSF project for the Restrain Food Database. The wiki section on the left-hand side provides a description of the project and the various components which comprise the project. The components can be found on the right-hand side of the page. Each of these have a web page similar to the format above with the wiki page detailing its contents. Relevant files and folders can be found in the Files section at the bottom left-hand corner of the web page.

The OSF project contains the complete database as well as the validated sample of images with associated ratings, and a dedicated R Shiny tool which can be used to search images based on the users' criteria. The main web page of this database is displayed in figure 1 and can be accessed on https://osf.io/7gcsk/. The OSF project is separated into various components which are briefly described below. Detailed descriptions as well as a list of files and folders can be found in the wiki tab of each component.

#### 2.2.1. Components

Various components of the project can be accessed from the main project page^1^ of OSF. The Protocol^2^ component contains our pre-registered study protocol for this investigation. The Restrain Food Database (full)^3^ contains the complete database of 4286 food images and The Restrain Food Database (validated)^4^ contains the set of 520 images used in the validation study and data generated during the investigation. Validation study materials^5^ contain raw data files and all R scripts used to summarize and analyse the validation study dataset.

### 2.3. R Shiny food database search app

We provide a dedicated R Shiny tool for aiding navigation at https://restrain.shinyapps.io/restrainfooddatabase/. Below is a brief overview of the tool and how it can be used to select images by applying filters for the associated descriptive information. A more detailed step-by-step tutorial can be found on the wiki of The Restrain Database (validated) component^6^.

**Figure 2.**
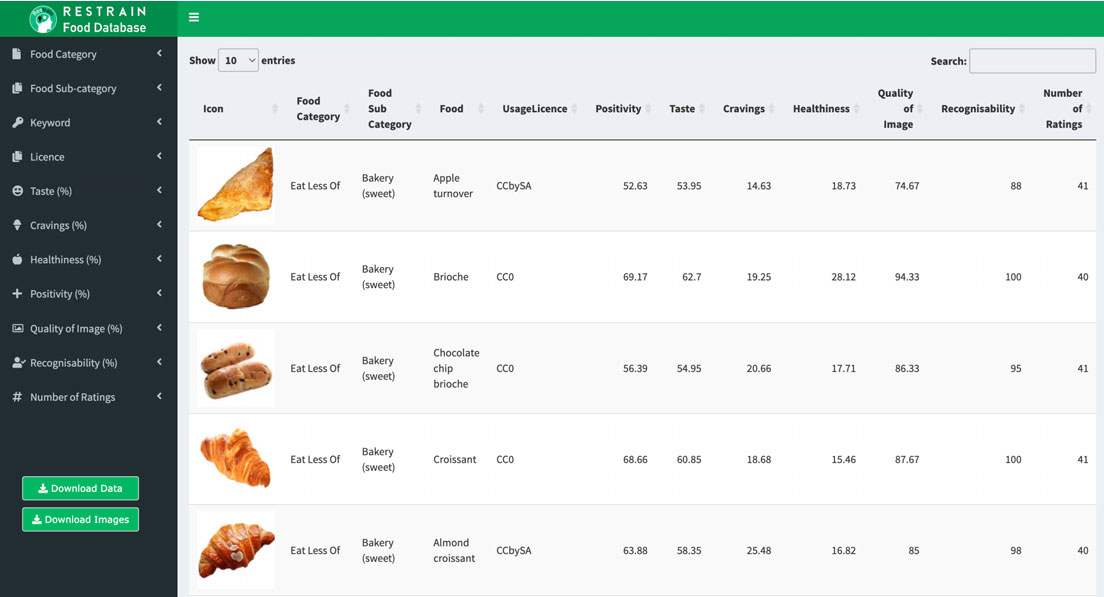
Image showing the homepage of the R Shiny search tool. The left-hand panel lists various filters that can be applied to produce a subset of the database. All filter options will be applied upon opening the web page. To filter the dataset, either untick the categorical filters, or drag slider filters to where appropriate. The database in the centre of the page displays the output of the current filters using an icon of the image as well as descriptive information. The subset of images can be downloaded by pressing ‘Download Images’ and the descriptive information (e.g. taste, craving ratings, nutritional categorization, etc.) can be downloaded in CSV format by pressing the ‘Download Data’ button in the bottom left of the screen.

Figure 2 shows an image of the web page of the R Shiny tool. All filters will be preset, meaning the data displayed in the centre of the screen is the complete validated database. To start filtering the database, click the filters on the left-hand panel. Categorical filters such as Food Category can be adjusted by using the tick boxes provided. Slider filters such as Recognisability can be applied by dragging the slider intervals to the appropriate values.

Once filters have been specified the subset of images can be downloaded as a .tar file by pressing ‘Download Images’. The associated descriptive data can be downloaded by pressing the ‘Download Data’ button in the bottom left-hand corner of the web page. This will produce a CSV containing descriptive information including quality ratings, subjective ratings and nutritional categorization for each individual item contained in the search filters.

In our validation study, only one out of seven exemplar images was used. Exemplar images only vary in regard to aspects such as the angle of the image or portion size, meaning generalization of subjective ratings across image exemplars may be appropriate. A more detailed step-by-step guide to navigating the R Shiny database can be found at https://osf.io/drvms/wiki/home/.

## 3. Validation study of database

### 3.1. Methods

#### 3.1.1. Design

**Figure 3.**
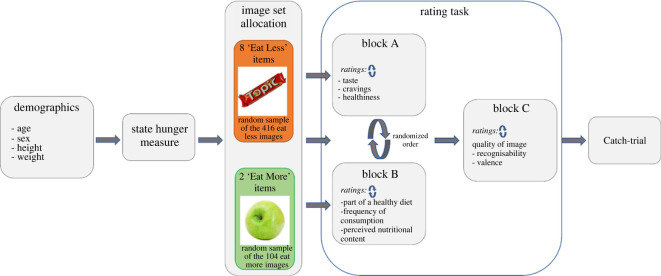
A schematic diagram of the study design. Upon opening the survey, participants provided consent followed by demographic details and completed a state hunger measure. Participants were randomly allocated into 1 of 52 between-group image sets. Each set consisted of eight ‘Eat Less’ and two ‘Eat More’ foods which were created by randomly assigning all images from the food groups to a set so that each image appeared only in one set. The rating task used a randomized block design for Block A and B with Block C being presented at the end. Participants rated all 10 food images in the set before they moved on to the next block. The presentation of images and order of rating measures were randomized within each block. Following Block C, a catch-trial was implemented as an attention-check with the order of response options being randomized. After completing the check, participants were provided debrief information. The study took approximately 12 min for participants to complete.

An online survey was conducted using Qualtrics (Provo, UT, 2022) with participants recruited through Prolific (Prolific, UK, 2022). Inclusion criteria were: aged 18+, currently residing in the UK, fluent in English, and consumes meat, dairy and fish products. Participants were not eligible to take part if they suffered from any major allergies. The study received ethical approval from Cardiff University Research Ethics Committee. A schematic diagram of the study design can be seen in figure 3.

The recruited sample used in our primary analyses consisted of 2150 participants. Descriptive statistics can be found in table 1. The sample had an average age of 42.09 (range = 18–83) and BMI of 26.90 kg m^−2^ (range = 20.98–58.42). It comprised 58.6% female participants and 82.3% identified as English, Welsh, Scottish, Northern Irish or British (a full breakdown of ethnic backgrounds can be found in table 2).

**Table 1.** Descriptive statistics of demographic variables of the sample.

demographic	mean (s.d.)	%
age	42.09 (13.91)	—
height (cm)	170.64 (9.55)	—
weight (kg)	78.62 (19.59)	—
BMI (kg m^−^^2^)	26.90 (6.98)	—
male	—	40.94
female	—	58.60
prefer not to say	—	0.47

**Table 2.** Count of ethnic backgrounds of the sample.

Ethnicity	*n*
African or Caribbean	58
Any other Asian, Black, African, Caribbean or other background	37
Any other mixed, multiple or white background	160
Arab, Bangladeshi, Chinese, Indian or Pakistani	65
English, Welsh, Scottish, Northern Irish, British or Irish	1783
White and Black African, Black Caribbean or Asian	47

#### 3.1.2. Procedure

Participants were randomly allocated into 1 of 52 between-person image sets which included 10 images consisting of two Eat More foods and eight Eat Less foods. These images were randomly selected from the subsample of total images used in the study.

After reading the study information, participants were asked to provide consent if they wished to take part. If consent was obtained, they provided demographic information (e.g. age, sex, ethnicity, height and weight), dietary status and requirements, and completed a state hunger measure. Participants then completed the rating task followed by an attention check (*identify the edible item in an array of images*) and were debriefed on the purpose of the study.

#### 3.1.3. Baseline demographics, dietary status and hunger rating

Participants’ age, sex, ethnicity, height and weight (which were used to calculate BMI) were obtained. Additionally, participants noted whether they suffered from any current and/or previous eating disorder diagnoses (*yes/no*), currently dietary status whereby they selected one of the following options: *‘I am actively trying to increase my food intake; I eat whatever I want, whenever I want; I often try to limit my food intake; I am constantly limiting my food intake’*. Participants also indicated if they had any dietary requirements or allergies (e.g. vegetarian, peanut allergy, etc.). Participants were not excluded if they noted that they have/had suffered from an eating disorder, but were if they indicated they had a dietary requirement or allergy as these factors may have an impact on ratings for individual food items.

Finally, participants were presented with a 100-point visual analogue scale (VAS) assessing current hunger (*How hungry do you feel right now?*) which was end-anchored ‘*not at all’* to *‘extremely’*.

#### 3.1.4. Stimuli

In the current validation study, we focused on UK foods only, therefore all images of soft drinks and non-UK foods were removed. All additional exemplar images were removed such that there was one image per food type. After removing these images, the subsample of food items used in the study consisted of 520 images (33 fruits, 71 vegetables, 86 sweet/savoury bakery, 45 savoury snacks, 102 biscuits/confectionery, 46 desserts, 137 takeaway) comprising 104 foods in the Eat More and 416 in the Eat Less category.

#### 3.1.5. Rating task

The ratings were presented in a block design in which liking, craving strength and perceived healthiness formed Block A, perceived category of either ‘Eat More’ or ‘Eat Less’, frequency of consumption, and perceived nutritional content formed Block B, and recognizability, quality and valence questions formed Block C.

Participants rated all 10 foods in each block before beginning the next block. Blocks A and B were presented in a randomized order with Block C always being presented last. This ordering meant that the blocks containing outcome measures relating to our primary measures were counterbalanced, with outcomes in Block C only used for image descriptive purposes.

**Figure 4.**
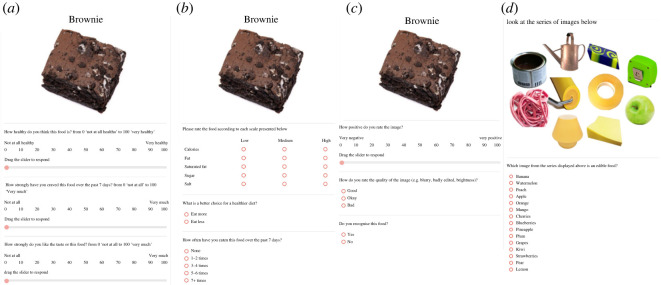
Panel showing an example of a trial from each block and the catch-trial. For the rating tasks, the image is presented in the middle of the screen with a description presented above. Rating measures are presented below the image in a randomized order. Panels (*a–c*) show the respectively named blocks; (*d*) shows the catch-trial used.

On the screen prior to beginning the rating task, participants saw the instructions ‘*You will be asked to rate a series of food images. It is important that you think about each question carefully, but not too long about your answers.’* Each image was presented in the centre of the screen against a white background with a brief description of the food item on top (figure 4).

*Block A measures*. Liking of taste *‘How strongly do you like the taste of this food?’* and cravings over the past 7 days *‘How strongly have you craved this food over the past 7 days?’* was measured on a 100-point VAS end-anchored *‘Not at all’* to *’Extremely’.* Perceived healthiness was also measured on a 100-point VAS end-anchored *‘Not at all healthy’* to *‘Very healthy’*.

*Block B measures*. Participants were asked ‘*What is a better choice for a healthier diet?*’ and chose between *‘Eat more’* or *‘Eat less’*. Frequency of consumption was assessed using a five-point scale: ‘*How often have you eaten this food over the past 7 days?*’ with options *‘None’*, *‘1–2 times’, ‘3–4 times’, ‘5–6 times’ and ‘7+ times’*. Perceived nutritional content was assessed by asking participants to rate the food as ‘*Low’, ‘Medium’ or ‘High’* in the following: calories, fat, saturated fat, sugar and salt.

*Block C measures*. Participants were asked to rate if they recognize the food presented in the image with a Yes/No response. Participants were also asked ‘*How do you rate the quality of the image (*e.g. *blurry, badly edited, brightness)?* With response options *‘Good’, ‘Okay’, ‘Bad’*. Valence of the image (i.e. ‘*How positive do you rate the image?*’) was assessed with a 100-point VAS end-anchored *‘Very negative’* to *‘Very positive’*.

*Attention check*. An attention check was employed immediately following Block C. This trial displayed an array of images and required the participant to select the edible food in the array of images (correct response being an apple). The response selection was in a randomized multi-choice format with 15 different food items to select from.

### 3.2. Data management and statistical analyses

#### 3.2.1. Attention check

The percentage of correct responses to the attention check is reported as a metric of the quality of data rather than excluding participants [26].

#### 3.2.2. Data exclusions

Pilot testing of the study was conducted on a small sample (*n* = 200) to determine any issues in the Prolific survey we created. This data was not included in analyses due to changes made to the survey (e.g. mislabelled food or response options). We used this data to determine the criteria for exclusion based on average time taken to complete one trial for each block. We determined participants who completed a Block A trial in less than 5 s, or a Block B or C in less than 4 s will be excluded (and replaced). We also excluded and replaced any participant who did not complete the study.

It was deemed necessary to exclude extreme cases in demographic variables due to the self-reported nature of these outcomes. Extreme cases for age (≥100), height (≤100 or ≥250), weight (≤40 or ≥600) and BMI scores (≤10 or ≥90) were excluded. We did not replace those excluded from demographic models as their data was maintained for all other forms of analyses.

#### 3.2.3. Descriptive statistics of food items

The primary purpose of this paper is to validate a sample of images from our open-source food image database and provide information on food images to aid researchers in determining suitable images to use in their own studies.

To this end, we provide means and standard deviations of ratings across Food Category (Eat More/Eat Less) as well as Food Subcategory (fruits, vegetables, sweet/savoury bakery, savoury snacks, biscuits/confectionery, desserts, generic/chain takeaway). We graphically represent these categorical differences between point estimates and their associated 95% confidence intervals using interval plots.

Frequency of weekly consumption is graphically represented on a bar chart broken down by Food Subcategory. We also provide the percentage of correct responses for perceived nutritional content. Correct responses were determined based on nutritional content codes provided for all foods within the study (low, medium or high, based on the Food Standards Agency's traffic light system described above). We also provide the percentage of correct responses for perceived eat more/eat less rating by Food Category. Finally, responses for image quality are provided as a percentage of responses per responses for both Food Categories.

Individual ratings for all 520 food items used in this validation study can be found in the file titled *‘The Restrain Food Database Catalogue Validated.csv’* on the Restrain Food Database (validated) (see footnote 4) component of the OSF website.

#### 3.2.4. Statistical analyses

Analyses were conducted using iterative generalized least squares (IGLS) bootstrapped (500 samples) multi-level modelling approach [27]. Models were created for Taste, Craving and Healthiness outcomes. These models described the proportion of variance which could be attributable to between-participant as well as within-trial differences.

For these outcomes, the dataset was structured so that trials were nested within participants, resulting in a two-level structure. A random intercept for each participant was created to provide better estimates of within-person estimates [28]. To ensure the appropriateness of including this random effect, the two-level null model was compared with a single-level model using a *χ*^2^ likelihood ratio test. This test was also used to assess model fit of conditional models against the two-level null models. If no significant variation was detected at the participant level, then single-level OLS models were used.

All between-participants variables (e.g. age, BMI, state hunger, etc.) were grand-mean centred [29]. Casewise deletion was performed for analyses with missing data present (i.e. Model 3).

Analyses were run in RStudio v. 1.4.1106 (RStudio Team, 2022) using R package *‘lme4’* [30]*.* The *α* level was set as < 0.05. The pre-registered protocol can be accessed at https://osf.io/m85sr/.

#### 3.2.5. Predictors of differences in ratings

We analysed various predictors of differences in ratings in our dataset by creating three separate models.

Model 1 investigated the strength of the difference between food categories (Eat More/Eat Less). Food category was entered into the model as a dummy variable with ‘Eat Less’ As the reference category.

Model 2 investigated the extent to which state hunger ratings were associated with outcomes. We included state hunger as a participant-level variable as well as the interaction term with Food Category to explore if categorical differences in ratings were greater or lesser in the context of higher hunger levels.

Model 3 investigated associations between demographic variables, age, sex and BMI which were all included in the model as participant-level variables. If any variables were identified as statistically significant, a model with an interaction term with Food Category was created to explore if between-person differences in ratings attributable to demographic variables resulted in greater or lesser differences between food categories. As we made no directional predictions of moderating effects of demographics, these were conducted only as exploratory follow-up on any main effects identified to avoid spurious results.

### 3.3. Deviations from pre-registration

#### 3.3.1. Association between average reaction time to trials and likelihood of providing a correct response to the catch-trial

In our pre-registered analyses, we set out to investigate whether the average speed of completing trials influenced the likelihood that a participant would correctly respond to the attention check. This analysis was planned to assess whether our catch-trial served as an accurate method of an attention check so that completion rates could be reported as a measure of data quality [26] However, very few participants responded incorrectly to the attention check, meaning there was insufficient variation in the outcome to perform this analysis.

#### 3.3.2. Contrasts of models between weight categories

A set of pre-registered primary analyses examining differences in primary outcomes between weight categories were redesignated as supplementary analyses. Concerns were raised in reviewer comments surrounding the appropriateness of making BMI a categorical instead of continuous predictor due to (i) a loss of information (within BMI category variance is lost); (ii) BMI weight categories are not based on well-justified thresholds. An additional problem with these analyses is that we neglected to pre-register a multiple comparison correction for these contrasts. No significant findings were identified after a Bonferroni adjustment was applied, which is in agreement with other analyses presented within this manuscript (i.e. BMI as a continuous predictor in Model 3).

## 4. Results

### 4.1. Descriptive statistics of food database

#### 4.1.1. Sample descriptives

The final dataset comprised 2150 participants. A total of 167 participants were excluded according to pre-registered criteria (19 for not completing the study and 148 for completing a trial in less than the block-specific cut-offs). Only 5 out of 2150 (0.23%) participants did not pass the attention check. Descriptive statistics of the sample are reported in table 1. Table 2 displays the ethnic background and table 3 displays the dietary status of the sample.

**Table 3.** Count of dietary status of the sample.

dietary status	*n*
constantly limit intake	110
often limit intake	1025
no restrictions	962
actively increasing food intake	53

#### 4.1.2. Trial descriptives

The final dataset consisted of 21 500 trials. We originally planned for equal group sizes (*n* = 41) across all sets; however, the final dataset demonstrated slight differences in these due to the randomization technique employed within Qualtrics. Out of the 52 between-person sets, there were six sets with *n* = 39, 10 sets with *n* = 40, 12 sets with *n* = 41, 14 sets with *n* = 42, five sets with *n* = 43, three sets with *n* = 44 and two sets with *n* = 45.

**Figure 5.**
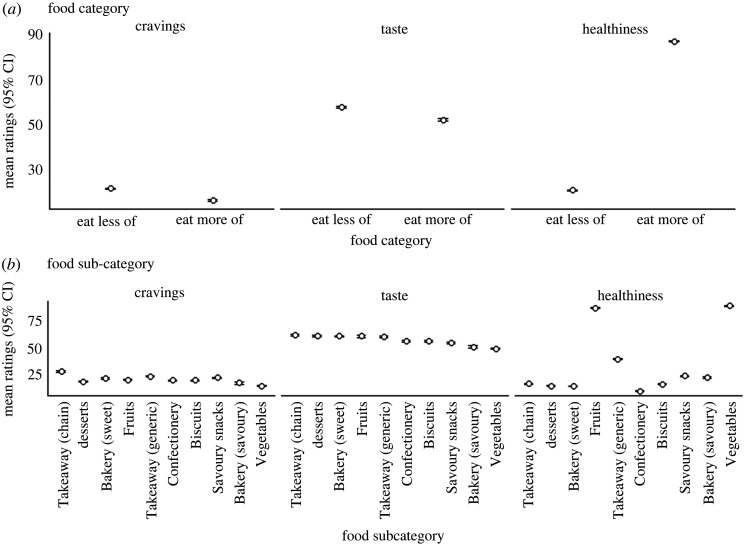
Interval plots showing mean responses for cravings, taste and healthiness ratings, and their associated 95% confidence intervals. (*a*) shows ratings at the Food Category level and (*b*) shows ratings at the subcategory level.

*Subjective ratings for food items.* Taste, Healthiness, and Craving ratings for Food Category and subcategories are displayed in tables 4 and 5 respectively. Estimates of mean ratings and their associated 95% confidence intervals broken down by Food Category and subcategory are displayed in figure 5.

**Table 4.** Means (s.d.) for outcomes by Food Category.

outcome	eat more	eat less
cravings	15.55 (23.83)	20.74 (27.28)
healthiness	86.43 (16.82)	20.03 (21.16)
taste	51.39 (32.62)	57.02 (32.85)

**Table 5.** Means (s.d.) for outcomes by Food Subcategory.

outcome	bakery (savoury)	bakery (sweet)	biscuits	confectionery	desserts	fruits	savoury snacks	takeaway (chain)	takeaway (generic)	vegetables
cravings	16.26 (22.74)	20.55 (27.10)	18.66 (24.91)	18.81 (27.02)	17.71 (25.37)	19.41 (26.75)	21.11 (28.33)	27.07 (30.42)	22.26 (27.29)	13.76 (22.13)
healthiness	21.58 (19.30)	13.43 (15.89)	15.44 (16.19)	8.95 (12.84)	13.57 (15.04)	85.00 (16.73)	22.68 (21.05)	15.57 (17.01)	38.47 (23.44)	87.09 (16.82)
taste	49.4 (32.77)	59.12 (32.38)	54.52 (31.46)	54.8 (34.27)	59.38 (32.50)	59.07 (32.41)	52.99 (34.03)	60.11 (31.97)	58.65 (31.89)	47.83 (32.11)

**Figure 6.**
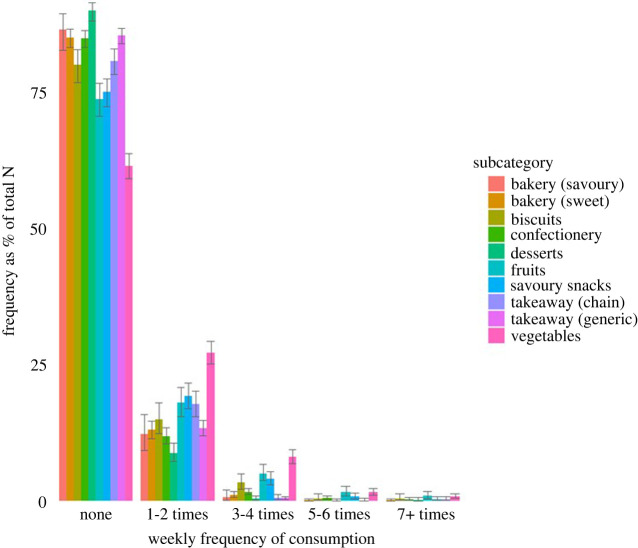
Bar chart describing responses for frequency of weekly consumption as the proportion of the responses within a subcategory. Ratings from each individual item in the study were counted according to their class interval (response categories for frequency of consumption) and subcategory. These frequencies were presented as the proportion of responses for a given class interval of the total amount of responses within a subcategory (i.e. approx. 20% of fruit items in this study were reported as being consumed 1–2 times a week across the sample). Error bars are 95% Wilson score intervals.

*Frequency of weekly consumption.* Figure 6 displays the frequency of weekly consumption for items with each bar representing a percentage of the total amount of items in each subcategory.

*Percentage of correct responses for perceived nutritional content.* Responses for the perceived nutritional content (i.e. fat, saturated fat, sugar and salt) were compared with the actual content of food items and this produced a percentage of correct responses for each nutritional value. The proportion of participants' judgements of food category to the databases categorization of foods produced a percentage of correct responses per Food Category (table 6).

**Table 6.** Proportion of correct responses for food category identification and nutritional content estimates. Values are % of correct responses determined by comparing responses with the actual values of each food.

outcome	eat more	eat less
correct category	89.19	92.54
fat content	47.09	93.75
saturated fat content	47.20	95.19
sugar content	69.30	72.55
salt content	47.57	91.89

*Ratings of image quality, recognizability and perceived valence.* Proportion of image quality ratings, proportion of items that were identified as being recognized, and how positive images were rated separated by Food Category are reported in table 7.

**Table 7.** Proportion of image quality response, recognizability of images and valence ratings. Values are % of responses provided.

outcome	eat more	eat less
good quality	69.62	50.83
okay quality	26.71	40.00
bad quality	3.68	9.17
recognizability	94.31	93.54
mean valence (s.d.)	68.67 (25.09)	56.27 (26.74)

### 4.2. Pre-registered statistical analyses

#### 4.2.1. Predictors of differences in subjective ratings

Table 4 for means and standard deviations of outcome measures.

Various analyses were conducted on the dataset presented in three separate models. We also performed sensitivity analyses as robustness checks, which can be found in electronic supplementary material. No qualitative differences were found between sensitivity analyses and the primary results reported in the main manuscript.

Model 1 investigated the main effect of Food Category. Model 2 investigated the influence of state hunger on ratings. Model 3 investigated the influence of demographic variables on ratings.

#### 4.2.2. Variance component models

To ensure the appropriateness of including participant as a random effect, two-level null models were compared with their single-level counterparts using a *χ*^2^ likelihood ratio test.

*Healthiness.* The two-level structure was not a better fit than the single-level model, $\chi_{1}^{2}\approx 0.001,$ *p* = 0.99. Therefore, ordinary least-squares linear regressions were used.

*Taste.* The two-level structure was a better fit than the single-level model, $\chi_{1}^{2}\approx 901.46,$ *p* < 0.001. An examination of the variance partition coefficients (VPCs) shows that 87.99% of unexplained variance was at the trial level, 12.02% was at the participant level.

*Cravings.* The two-level structure was a better fit than the single-level model, $\chi_{1}^{2}\approx 2570.21,$ *p* < 0.001. An examination of the VPCs shows that 77.41% of unexplained variance was at the trial level, 22.59% was at the participant level.

#### 4.2.3. Differences in subjective ratings between food category (model 1)

To assess the extent that subjective ratings differed between Food Categories (Eat More/Eat Less), Food Category was entered as a trial-level dummy variable (reference category = Eat Less).

*Healthiness.* Adjusting for Food Category resulted in a better fit to the model and explained 62.99% of the variance (_adj_*R*^2^ = 0.630, *F*_(__1, 21498)_ = 3659.0, *p* < 0.001). Healthiness ratings were significantly higher for Eat More compared with Eat Less items (table 8).

**Table 8.** Ordinary least squares regression models for Healthiness ratings.

	*B*	s.e.	95% CI	*p*
M1				
food category	66.40	0.347	65.72 67.08	*<0.001*
M2				
food category	66.40	0.347	65.72 67.08	*<0.001*
hunger	0.043	0.006	0.031 0.056	*<0.001*
food category × hunger	−0.037	0.014	−0.065–0.009	*0.009*
M3				
age	0.008	0.017	−0.025 0.041	0.617
sex (male)	0.462	0.471	−0.461 1.39	0.326
BMI	−0.073	0.038	0.148 0.002	0.055

*Taste.* Including Food Category improved the model fit. A comparison of the VPCs indicated including the predictor explained 0.91% at the trial level, but increased the unexplained variance by 0.12% at the participant level. Taste ratings were significantly lower for Eat More compared with Eat Less items (table 9).

**Table 9.** Random intercept linear models for Taste ratings.

		B	s.e.	95% CI	*p*
M1					
likelihood ratio *χ*^2^	115.13				*<0.001*
food category		−5.63	0.524	−6.75 −4.56	*<0.001*
M2					
likelihood ratio *χ*^2^	149.04				*<0.001*
food category		−5.63	0.524	−6.62 −4.66	*<0.001*
hunger		0.075	0.014	0.047 0.104	*<0.001*
food category × hunger		−0.081	0.021	−0.126 −0.033	*<0.001*
M3					
likelihood ratio *χ*^2^	7.01				*0.071*
age		−0.032	0.024	−0.079 0.015	*0.174*
sex (male)		1.31	0.667	0.030 2.62	*0.049*
BMI		0.085	0.054	−0.026 0.184	*0.114*

*Cravings.* Including Food Category improved the model fit. Comparing the VPCs of the null with the predictor model revealed that including the predictor explained 0.91% at the trial level, and unexplained variance increased by 0.26% at the participant level. Craving ratings were significantly lower for Eat More relative to Eat Less (table 10).

**Table 10.** Random intercept linear models for Craving ratings.

		B	s.e.	95% CI	*P*
M1					
likelihood ratio *χ*^2^	*168.74*				*<0.001*
food category		−5.19	0.399	−5.99 −4.40	*<0.001*
M2					
likelihood ratio *χ*^2^	*286.27*				*<0.001*
food category		−5.19	0.399	−5.96 −4.37	*<0.001*
hunger		0.141	0.013	0.115 0.168	*<0.001*
food category × hunger		−0.075	0.016	−0.108 −0.043	*<0.001*
M3					
likelihood ratio *χ*^2^	*30.93*				*<0.001*
age		−0.119	0.23	−0.162 −0.074	*<0.001*
sex (male)		1.78	0.648	0.367 3.13	*0.006*
BMI		0.004	0.052	−0.101 0.100	*0.936*

#### 4.2.4. Impact of state hunger levels on subjective ratings of food images (model 2)

To assess the impact of participants’ current level of hunger on ratings, we included state hunger ratings as a participant-level variable to account for between-person differences in ratings that could be attributable to participants' current levels of hunger. We also included the interaction term between state hunger and food category to assess whether differences in ratings between food categories were greater or lesser during high levels of hunger.

*Healthiness.* Adjusting for predictors resulted in a better fit to the model and explained 63.07% of the variance (_adj_*R*^2^ = 0.631, *F*_(__3,21498)_ = 1224.0, *p* < 0.001).

Table 8 for results. In summary, Healthiness ratings were significantly higher for Eat More relative to Eat Less items. A higher rating on the state hunger measure was also associated with a greater between-person average Healthiness ratings.

**Figure 7.**
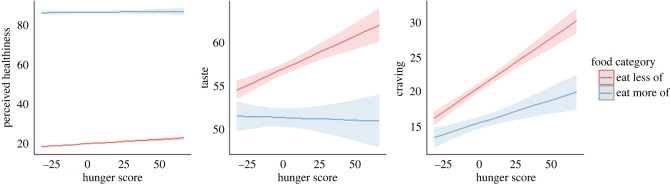
Interaction plots of hunger and food category for primary outcomes. Shading around lines indicates 95% confidence intervals. Note that hunger score has been mean-centred.

An interaction effect was also identified. Individuals who reported higher levels of hunger displayed lower levels of differences in Healthiness ratings between food categories (i.e. the more hungry a participant reported as being, the more similar their healthiness ratings were between food categories) (figure 7).

*Taste.* Including predictors significantly improved the model fit. A comparison of the VPCs indicated including the predictor explained 0.38% at the participant level, but increased the unexplained variance by 0.05% at the trial level.

Table 9 for results. In summary, Taste ratings were significantly lower for Eat More relative to Eat Less items. A higher state hunger rating was associated with greater between-person average Taste ratings.

The interaction term was also significant. Individuals who reported higher levels of hunger displayed further differences in Taste ratings between Eat More and Eat Less foods (figure 7).

*Cravings.* Including predictors significantly improved the model fit. Comparing the VPCs of the null with the predictor model revealed that adjusting for predictors explained 3.51% at the participant level, but increased the unexplained variance by 1.05% at the trial level.

In summary, Craving ratings were significantly lower for Eat More relative to Eat Less items (table 10). An increase in state hunger was also associated with higher between-person average craving ratings.

The interaction term was also found to be significant. Individuals who reported higher levels of hunger displayed greater differences in craving ratings between food categories (figure 7).

#### 4.2.5. Demographic between-person differences in average subjective ratings (model 3)

To assess whether any demographic variables are associated with between-person differences in ratings, we included Age, Sex (reference category = female), and BMI as predictors.

Casewise exclusion was performed on missing data for BMI (*n* = 6) or Age (*n* = 0) due to data exclusions for extreme cases. We also excluded cases where Sex was reported as ‘Prefer not to say’ (*n* = 1). The subset of data for these analyses consisted of 2134 participants.

Adjusting for predictors did not improve the fit of the model for Healthiness (_adj_*R*^2^ < 0.001, *F_(_*_3,21336)_ = 1.62, *p* = 0.182) or Taste ratings (table 9), but did so for craving ratings. Comparing the VPCs of the null with the predictor model revealed that adjusting for predictors explained 1.51% in variance of craving ratings at the participant level, but marginally increased the unexplained variance by 0.004% at the trial level (table 10).

In summary, increased age was associated with lower between-person average for craving ratings. Participants reporting as being male (relative to female) was associated with increases in between-person average of craving ratings.

**Figure 8.**
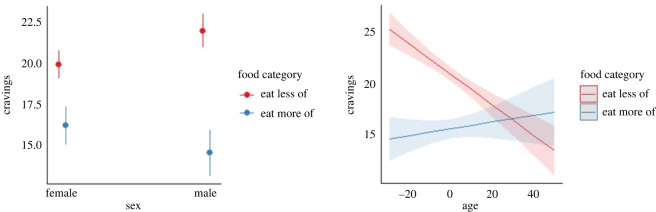
Interaction plots of sex and food category for cravings on the left hand panel with lines denoting 95% intervals. The right hand panel is an interaction plot of age and food category with shading around lines indicating 95% confidence intervals. Note that age has been mean-centred.

An interaction model was created where separate terms were included for Age and Sex with their interaction of Food Category. The interaction terms for Age (*β* = 0.020 (0.029), 95% CI = 0.140 to 0.254, *p* < 0.001) and Sex (*β* = −4.49 (0.822), 95% CI = −6.17 to −2.80, *p* < 0.001) were statistically significant (figure 8).

Participants reporting as being male (relative to female) demonstrated greater differences between Eat More and Eat Less items. Additionally, increases in age were associated with lower levels of difference in craving ratings between Eat More and Eat Less foods.

## 5. Discussion

The aim of this study was to validate and release an open-source food image database on measures relating to appetite, perceived nutritional content and image quality to confirm our categorization of foods that individuals intend to eat more or less of as part of a healthier diet. We provide data concerning categorical differences on the various measures employed from 2150 participants. In doing so, we achieved our secondary goal to provide descriptive information that could be used by future researchers to help inform decisions regarding stimuli selection for their own investigations. The Restrain Food Database thus complements and extends existing resources such as the Food-pics databases or the more recent CROCUFID [17,19,20].

Results of our online study confirmed that food items which were classified as Eat More were perceived as more healthy compared with Eat Less foods, reaching an approximately 66-point difference between categories on a 100-point VAS rating scale. An examination of the differences between food subcategories displayed in figure 5 demonstrates fruits and vegetables are rated similar on perceived healthiness. Eat Less subcategories were also rated similarly with one exception: takeaway (generic) items appeared to be rated healthier than other subcategories in this domain. A potential explanation for this discrepancy may be that perceptions of healthiness could be influenced by the fact many of the recipes in this subcategory are easily homemade and could be modified to be healthier. Home-cooked meals are commonly perceived as healthier than ready meals, though in reality, nutrient and calorie content display similar levels [31].

As expected by previous studies [4] we also demonstrated that Eat More foods were rated lower on taste and craving relative to Eat Less foods, although these effects were less pronounced. The inclusion of Food Category explained low levels of variance (less than 1% of within-trial variance for both outcomes) suggesting there are other factors which may explain greater amounts of between-item differences in ratings. For example, accounting for food preferences may explain more variation as higher ratings for taste and cravings would be expected for the specific foods that participants prefer. Participants in our study were shown a random sample of images from a diverse range of food, meaning many items may not be preferred or even disliked.

Another influence of the small effects may be differences in craving and taste ratings between subcategories of Eat More items. Vegetables were rated as having the lowest levels of cravings and taste, whereas ratings for fruit were more consistent with the levels reported for Eat Less subcategories, supporting previous studies [5]. Nutritional aspects of fruit (e.g. high sugar content) may make this subcategory more desired and liked, whereas vegetables tend to lack these nutritional qualities. Another noteworthy observation regarding subcategory differences is that Takeaway (chain) was craved the most and received the highest taste ratings. This could have been influenced by widespread familiarity with branded fast food chains. Future investigations using this open database could explore subcategory differences in branded versus unbranded items.

Our categorization of foods as Eat More/Eat Less as part of a healthy diet is one of potential debate. Simple dichotomies do not capture all the similarities within a group and differences between them (e.g. cheese or yoghurt can be considered healthy as part of a balanced diet). However, grouping food into categories is of practical need for food-related research. We validated our categorization based on widely used techniques for assessing nutritional quality (i.e. NHS 5-a-day and the Food Standards Agency's traffic light system) which, crucially, are familiar methods for members of the public. Public understanding was important for our validation study and the wider Restrain Trial, as both are online studies lacking researcher oversight in which opportunities to explain more nuanced categorizations are not available.

Gibbons *et al*. [13] highlighted the importance of accounting for individual differences in appetitive outcomes, making multi-level modelling well suited for the rating measures used in this study due to the ability to quantify the proportion of variance in outcomes that could be attributable to within- and between-person differences. The variance component models revealed significant between-person variation in both taste and craving ratings, meaning some individuals rated that they craved or liked the taste of items more (or less) than the sample average. Notably, no multi-level structure was identified in perceived healthiness ratings. In the context of the high proportion of correct responses for perceived food category of items, and inclusion of Food Category explaining approximately 63% of variance, a lack of between-person differences in perceived healthiness demonstrates high between-person agreement in the healthiness of items within these categories. It is important to note that main and interactive effects of between-person predictors were all small. The largest effect of hunger identified in our analyses was on craving ratings (*β* = 0.141).

Baseline state hunger ratings were associated with differences on our subjective rating measures. Higher levels of state hunger was associated with greater than average between-person levels on all our subjective rating measures. State hunger ratings also appeared to moderate the effect of Food Category on these outcomes. For taste and craving ratings, Eat More items were rated lower than Eat Less items, and individuals who reported greater levels of hunger demonstrated greater differences in ratings between food categories. For healthiness ratings, Eat More items were rated higher than Eat Less items with individuals reporting greater levels of hunger demonstrating lower levels of differences between healthiness ratings of Food Categories.

While our measure of cravings related to experiences over the past 7 days, state hunger asked how participants felt in that moment. However, this association may be unsurprising given the link between these two appetitive processes [11]. Heightened levels of hunger may make recall of cravings for foods more available [12]. Alternatively, raised hunger may make participants more reactive toward the rewarding qualities of the food items which could have influenced craving ratings [1,2].

Evidence was also obtained for between-person demographic differences in ratings on craving outcomes. Male relative to female participants reported higher between-person levels of cravings. This finding is in contrast to previous investigations which have either found female participants have increased levels of food cravings relative to male participants [8,9] or those that failed to find an effect [10]. One potential explanation for this is due to differences in methodologies used to measure cravings. Previous studies have used trait craving inventories which ask participants to reflect upon cravings they experienced for specific food groups. We assessed cravings by aggregating ratings across food items, many of which may be unfamiliar to participants. We found an interactive effect between sex and Food Category demonstrating male participants reported even greater differences in cravings between food categories relative to female participants. This evidence provides support for previous studies demonstrating sex-related difference in cravings exist between different types of food [6].

Increased age was also associated with lower between-person levels of cravings, supporting previous studies [7]. Additionally, increased age was associated with lower levels of differences in cravings between food categories. This suggests that as individuals age, cravings for foods that are generally regarded as being healthy increase, while craving for unhealthy foods decrease. We did not find any significant association between cravings and BMI, which does not support previous studies [6], though it must be stated that we measured the intensity of cravings whereas others measured their frequency, which may explain the disparity in these findings.

Our investigation also revealed that the vast majority of food items were not consumed or were only consumed between one and two times a week. One factor which may have influenced the low rates might be that participants were shown a random selection of a diverse array of 520 food items.

Participants appeared to be better at correctly identifying the nutritional content of Eat More (between 72 and 95% of correct responses) compared with Eat Less (between 47 and 69% of correct responses) foods.

The majority of the quality of images within the study were rated as either ‘good’ or ‘okay’, with low rates of ‘bad’ images for each category. The valence of images was also rated as generally positive. These descriptive statistics demonstrate that there are no systemic issues with quality or the usability of images within our database on average. However, these factors are more informative and have more practical implications at the image level. As discussed above, ratings for each individual image can be found on the OSF project's web page and can be applied as filters in the associated R Shiny app.

Toet *et al*. [17] noted previous food image databases suffer from one or more of the following issues: (i) they only provide a limited selection of Western foods; (ii) they are not standardized across image characteristics (i.e. background, colour, brightness, size, etc.); (iii) they do not provide normative data such as measures of valence, image quality or appetite-specific ratings (e.g. taste and cravings). While the Restrain Food Database complements and extends upon existing resources, it does not address all these existing issues, which are discussed below. Nonetheless, it is unlikely that one single database can address all of these concerns, but using multiple databases in combination with each other is a pragmatic solution. It is also worth mentioning that there is scope for our database to be extended upon through future add-ons of food images, which would be particularly useful for image banks of non-Western foods.

## 6. Strength and limitations

One major advantage of this food image database over existing databases is that it provides a greater range of food categories comprising multiple exemplar images for each item, offering the opportunity to provide participants a large selection of foods for them to choose. This is advantageous because it addresses a large practical issue in investigations of food-related behaviours when creating appropriate standardized images for stimuli, particularly for personalized stimuli.

An additional strength of this study was that findings were yielded from a large sample of participants. This led to a large number of ratings for each individual item within the study (*n* = 39–45) resulting in greater confidence in the estimates yielded both at the Food Category and individual-item level. Finally, our database is the first to provide a dedicated search tool to aid with navigating the food database. By utilizing the normative data generated within this study, individuals using our database can use the R Shiny tool to set filters to gain a subset of food images based on their desired criteria.

One limitation of this investigation is that we validated only a sample of the food images provided within the database, meaning some food items within our database lack validation and descriptive information. For example, only one of seven exemplar images for each of the included foods was used. However, exemplar images are visually similar to one another, meaning it may be safe to generalize ratings across exemplars. Nonetheless, it should be noted that we excluded non-UK foods and sugary drinks, and only recruited individuals who are currently residing in the UK and have no dietary requirements (e.g. suffer from allergies or report as not eating specific food groups). In the light of these limitations, we caution these findings may suffer from issues relating to a lack of cross-cultural validation as well as generalization across individuals with different dietary requirements.

Another limitation was that our study has a heavy reliance on self-reported data due to the online survey method used. For example, height and weight were self-reported and we employed no means of validating these responses, which could have had an impact on the validity of results surrounding BMI and weight category analyses.

A central issue in the data quality of online research conducted through platforms such as Prolific or Qualtrics is the monetary incentive that these services provide. Participants are motivated to maximize income by engaging in a large list of surveys available. The availability of surveys seems a likely drive to minimize the time and effort spent on any one survey in order to maximize income across many. We attempted to account for this with strict data exclusion criteria where participants who completed just one trial faster than the block-specific cut-offs were excluded. However, no attempts will completely solve the inherent data quality problem for research conducted through online platforms where financial incentive is primarily driven through completing as many different surveys as possible.

Finally, only a low proportion of individuals failed the attention check (approx. 0.23%) suggesting the possibility of a ceiling effect. For this reason, we could not conduct our pre-planned analysis examining how average reaction time to complete trials influenced the likelihood that a correct response to the catch-trial was provided meaning this measure was not viable as a metric of data quality. A further issue is that we did not exclude participants who failed the attention check in our final dataset (*n* = 5). Given the ease of the task and the low failure rates reported, it is likely these cases may be of low quality. However, no substantive differences were found during our sensitivity analyses. Furthermore, multi-level analyses account for variance in trials accountable to differences in participant averages, and given that these analyses were highly powered, the effect of these cases would be negligible.

## 7. Summary

In summary, this study validates the Restrain Food Database, including our predefined categorization of food items as those that individuals may wish to eat more or less of as part of a healthier diet. Descriptive information confirms sufficient levels of usability and image quality. This open-source resource is among the first to provide a wide variety of food items as well as descriptive information and a dedicated search tool with the intended purpose to aid decisions for stimuli selection when designing studies. The tool should be particularly effective in designs accounting for stimulus differences (e.g. selecting items rated high on taste or craving) and individual differences by employing personalization of choice among a diverse array of food items. It is our goal for this database to become a useful resource for potential researchers by reducing the burden of creating stimuli.

## Data Availability

Our main OSF web page can be found at https://osf.io/7gcsk/, which signposts relevant components such as the food image database. Data, study materials and analyses scripts for this validation study can be found on https://osf.io/wsqyn/. Our pre-registered study protocol can be found on https://osf.io/m85sr/. The data are provided in electronic supplementary material [32].

## Footnotes

1
https://osf.io/7gcsk/

2
https://osf.io/m85sr/

3
https://osf.io/czpxk/

4
https://osf.io/drvms/

5
https://osf.io/wsqyn/

6
https://osf.io/drvms/wiki/home/

## References

[1] Boswell RG, Kober H. 2016 Food cue reactivity and craving predict eating and weight gain: a meta-analytic review. Obes. Rev.: Off. J. Int. Assoc. Study Obes. **17**, 159-177. (10.1111/obr.12354) PMC6042864 26644270

[2] Dagher A. 2012 Functional brain imaging of appetite. Trends Endocrinol. Metab. **23**, 250-260. (10.1016/j.tem.2012.02.009) 22483361

[3] Mozaffarian D, Hao T, Rimm EB, Willett WC, Hu FB. 2011 Changes in diet and lifestyle and long-term weight gain in women and men. N. Engl. J. Med. **364**, 2392-2404. (10.1056/NEJMoa1014296) 21696306 PMC3151731

[4] Mela DJ. 2006 Eating for pleasure or just wanting to eat? Reconsidering sensory hedonic responses as a driver of obesity. Appetite **47***,* 10-17. 16647788 10.1016/j.appet.2006.02.006

[5] Meule A. 2020 The psychology of food cravings: the role of food deprivation. Curr. Nutr. Rep. **9**, 251-257. (10.1007/s13668-020-00326-0) 32578025 PMC7399671

[6] Chao A, Grilo CM, White MA, Sinha R. 2014 Food cravings, food intake, and weight status in a community-based sample. Eat. Behav. **15**, 478-482. (10.1016/j.eatbeh.2014.06.003) 25064302 PMC4115250

[7] Abdella HM, El Farssi HO, Broom DR, Hadden DA, Dalton CF. 2019 Eating behaviours and food cravings; influence of age, sex, BMI and FTO genotype. Nutrients **11**, 377. (10.3390/nu11020377) 30759834 PMC6412354

[8] Cepeda-Benito A, Fernandez MC, Moreno S. 2003 Relationship of gender and eating disorder symptoms to reported cravings for food: construct validation of state and trait craving questionnaires in Spanish. Appetite **40**, 47-54. (10.1016/S0195-6663(02)00145-9) 12631504

[9] Lafay L, Thomas F, Mennen L, Charles MA, Eschwege E, Borys JM. 2001 Gender differences in the relation between food cravings and mood in an adult community: results from the Fleurbaix Laventie Ville Sante study. Int. J. Eat. Disord. **29**, 195-204. 11429982 10.1002/1098-108x(200103)29:2<195::aid-eat1009>3.0.co;2-n

[10] Klimesova I, Elfmark M, Stelzer J. 2020 Food craving intensity and gender differences. Am. J. Health Educ. **51**, 179-185. (10.1080/19325037.2020.1744489)

[11] May J, Andrade J, Kavanagh DJ, Hetherington M. 2012. Elaborated intrusion theory: a cognitive-emotional theory of food craving. Curr. Obes. Rep. **1**, 114-121.

[12] Berry LM, Andrade J, May J. 2007 Hunger-related intrusive thoughts reflect increased accessibility of food items. Cogn. Emot. **21**, 865-878. (10.1080/02699930600826408)

[13] Gibbons C, Hopkins M, Beaulieu K, Oustric P, Blundell JE. 2019 Issues in measuring and interpreting human appetite (satiety/satiation) and its contribution to *obesity**.* Curr. Obes. Rep. **8**, 77-87. (10.1007/s13679-019-00340-6) 31037612 PMC6517339

[14] Field M, Werthmann J, Franken I, Hofmann W, Hogarth L, Roefs A. 2016 The role of attentional bias in obesity and addiction. Health Psychol. **35**, 767. (10.1037/hea0000405) 27505196

[15] van Ens W, Schmidt U, Campbell IC, Roefs A, Werthmann J. 2019 Test-retest reliability of attention bias for food: robust eye-tracking and reaction time indices. Appetite **136**, 86-92. (10.1016/j.appet.2019.01.020) 30682381

[16] Masterton S, Hardman CA, Halford JC, Jones A. 2021 Examining cognitive bias modification interventions for reducing food value and choice: two pre-registered, online studies. Appetite **159**, 105063. (10.1016/j.appet.2020.105063). 33279528

[17] Toet A, Kaneko D, de Kruijf I, Ushiama S, van Schaik MG, Brouwer AM, Kallen V, van Erp JBF. 2019 CROCUFID: a cross-cultural food image database for research on food elicited affective responses. Front. Psychol. **25**, 10-58. (10.3389/fpsyg.2019.00058) PMC6355693 30740078

[18] Foroni F, Pergola G, Argiris G, Rumiati RI. 2013 The FoodCast Research Image Database (FRIDa). Front. Hum. Neurosci. **751**, 51. (10.3389/fnhum.2013.00051) PMC3585434 23459781

[19] Blechert J, Meule A, Busch NA, Ohla K. 2014 Food-pics: an image database for experimental research on eating and appetite. Front. Psychol. **5**, 617. (10.3389/fpsyg.2014.00617) 25009514 PMC4067906

[20] Blechert J, Lender A, Polk S, Busch NA, Ohla K. 2019 Food-Pics_Extended—an image database for experimental research on eating and appetite: additional images, normative ratings and an updated review. Front. Psychol. **10**, 307. (10.3389/fpsyg.2019.00307) 30899232 PMC6416180

[21] Miccoli L, Delgado R, Rodríguez-Ruiz S, Guerra P, García-Mármol E, Fernández-Santaella MC. 2014 Meet OLAF, a good friend of the IAPS! The open library of affective foods: a tool to investigate the emotional impact of food in adolescents. PLoS ONE **9****,** e114515. (10.1371/journal.pone.0114515) 25490404 PMC4260831

[22] Miccoli L, Delgado R, Guerra P, Versace F, Rodríguez-Ruiz S, Fernández-Santaella MC. 2016 Affective pictures and the Open Library of Affective Foods (OLAF): tools to investigate emotions toward food in adults. PLoS ONE **11**, e0158991. (10.1371/journal.pone.0158991) 27513636 PMC4981440

[23] Charbonnier L, van Meer F, van der Laan LN, Viergever MA, Smeets PAM. 2016 Standardized food images: a photographing protocol and image database. Appetite **96**, 166-173. (10.1016/j.appet.2015.08.041) 26344127

[24] NHS. 2018 *5 A Day:* *what counts?* See https://www.nhs.uk/live-well/eat-well/5-a-day/5-a-day-what-counts/ (accessed 28 June 2022).

[25] Food Standard Agency. 2020 *Check the label*. See https://www.food.gov.uk/safety-hygiene/check-the-label (accessed 28 June 2022).

[26] Anduiza E, Galais C. 2017 Answering without reading: IMCs and strong satisficing in online surveys. Int. J. Public Opin. Res. **29**, 497-519.

[27] Rasbash J, Steele F, Browne WJ, Goldstein H. 2019 A user‘s guide to MLwiN, version 3.03. Bristol, UK: Centre for Multilevel Modelling, University of Bristol.

[28] Enders CK, Tofighi D. 2007 Centering predictor variables in cross-sectional multilevel models: a new look at an old issue. Psychol. Methods. **12**, 121-138. 17563168 10.1037/1082-989X.12.2.121

[29] Kreft IGG, Leeuw J, Aiken LS. 1995 The effect of different forms of centering in hierarchical linear models. Multivar. Behav. Res. **30**, 1-21. 10.1207/s15327906mbr3001_1 26828342

[30] Bates D, Mächler M, Bolker BM, Walker SC. 2015 Fitting linear mixed-effects models using lme4. J. Stat. Softw. **67**, 1-48.

[31] Naruseviciute G, Whybrow S, Macdiarmid JI, McNeill G. 2015 Is ‘home cooked’ healthier and cheaper than ready meals? Proc. Nutr. Soc. **74**, E90. (10.1017/S0029665115001056)

[32] Randle M, Duarte I, Maizey L, Tzavella L, Adams RC, Chambers CD. 2022 The Restrain Food Database: validation of an open-source database of foods that should be eaten more or less as part of a healthy diet. Figshare. (10.6084/m9.figshare.c.6296363) PMC9682305 36425519

